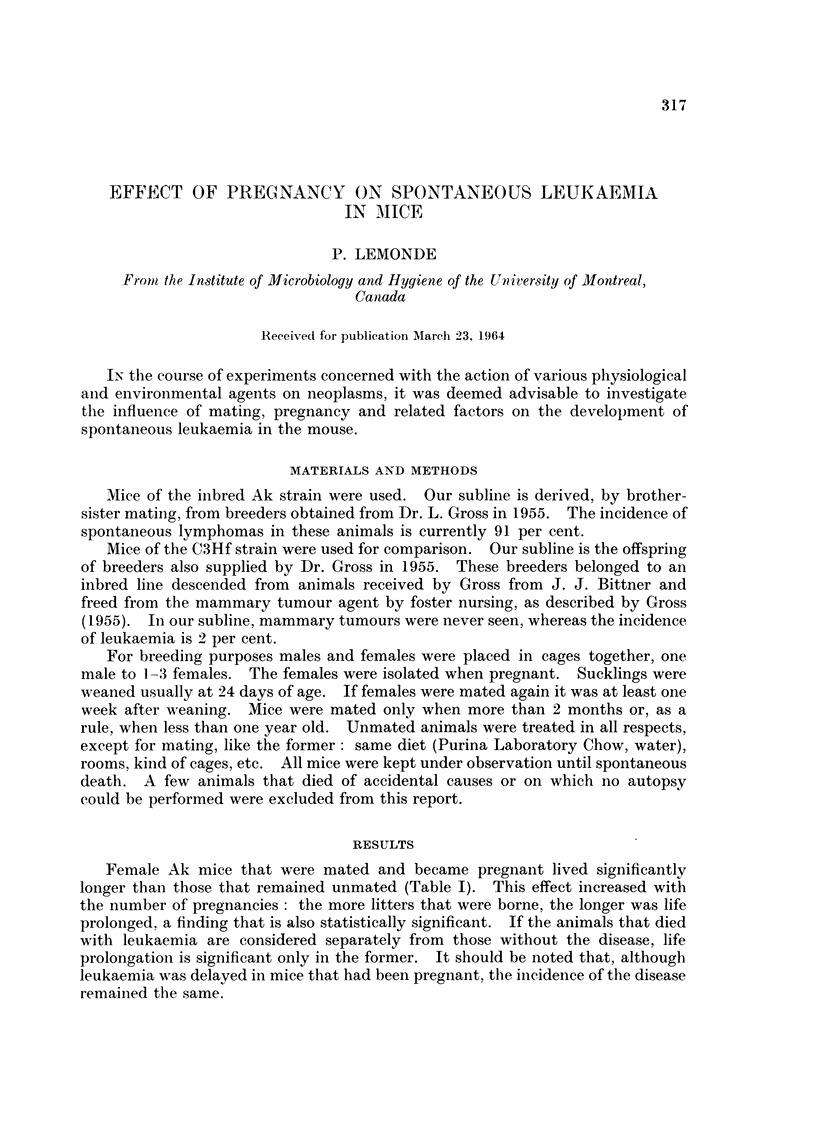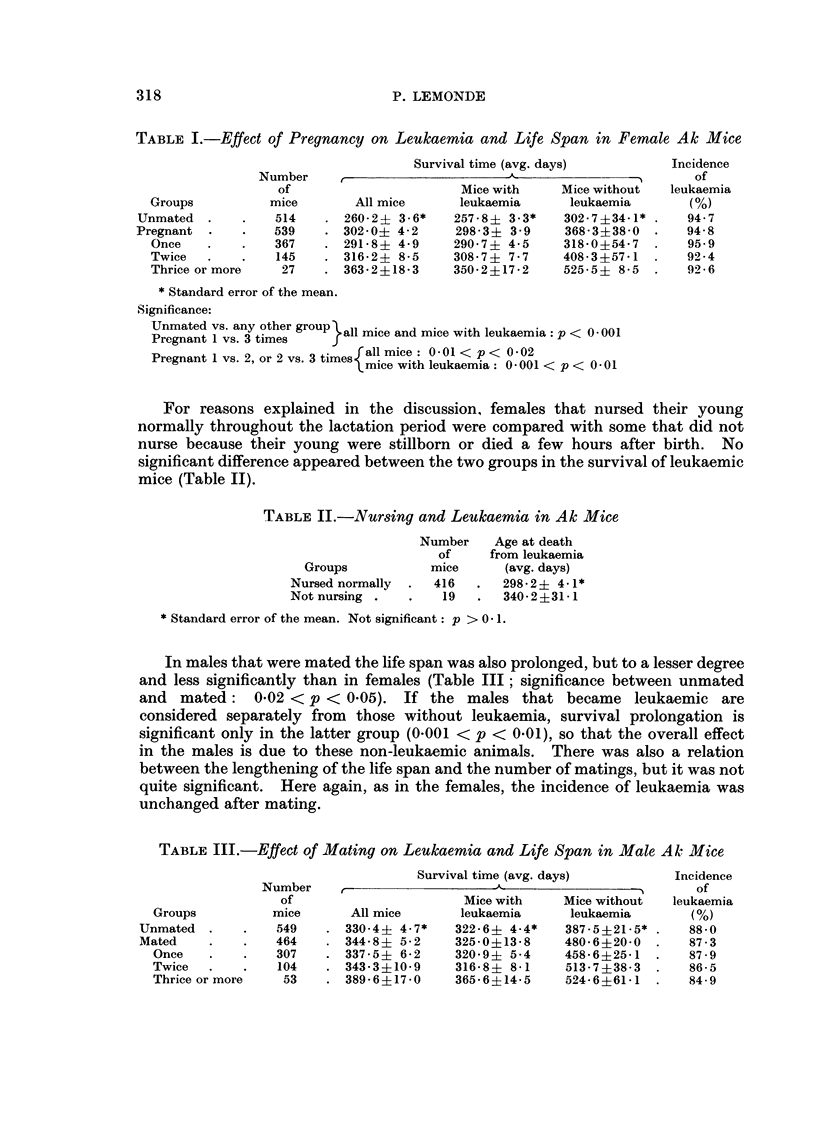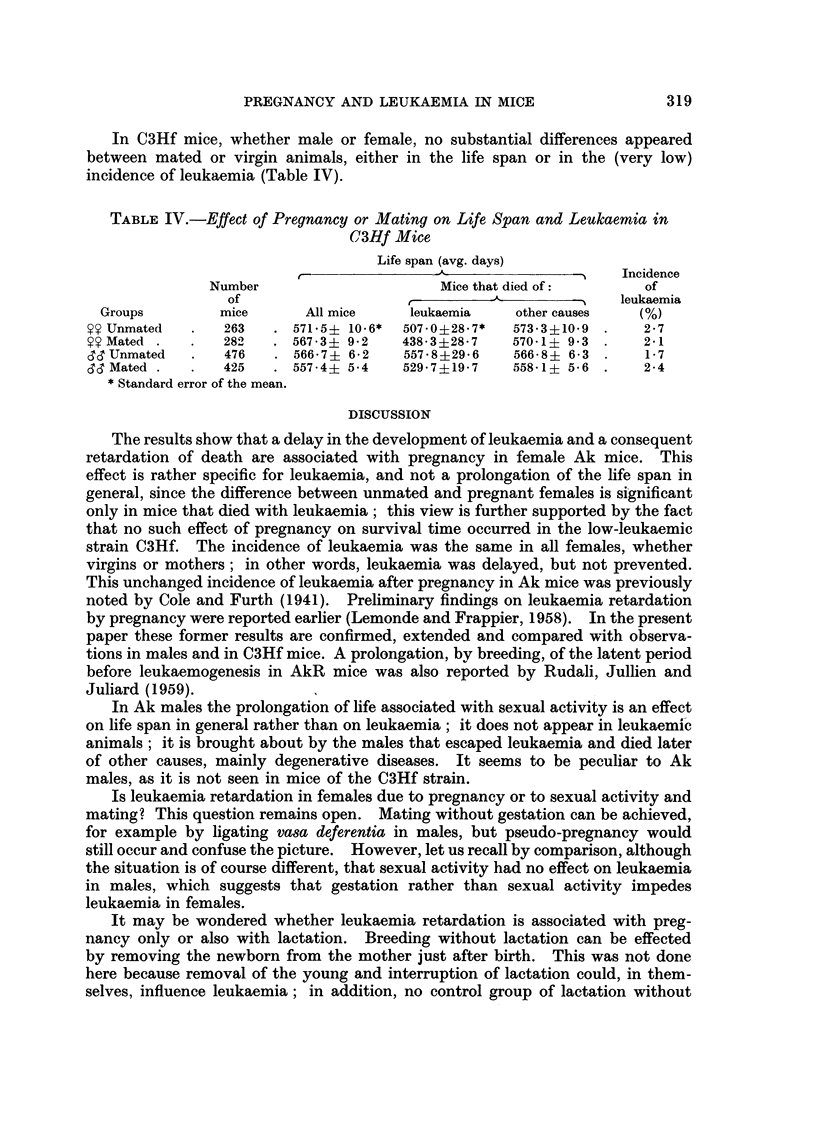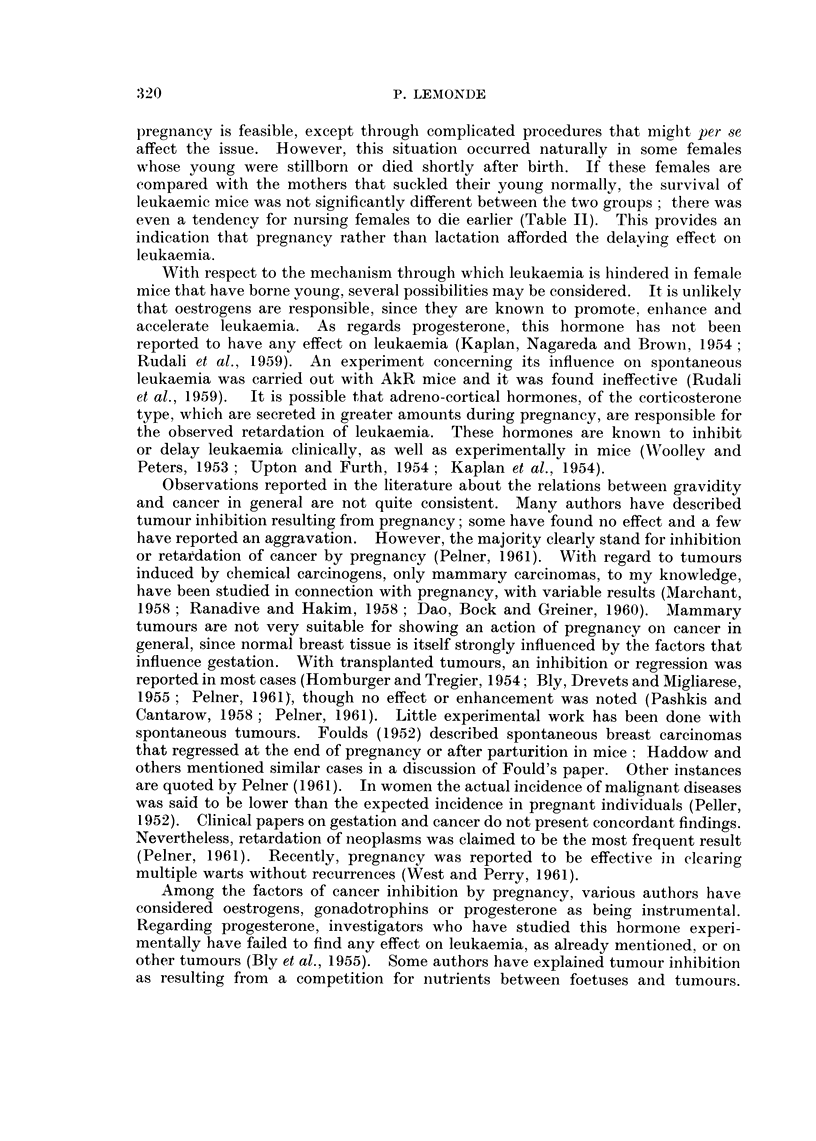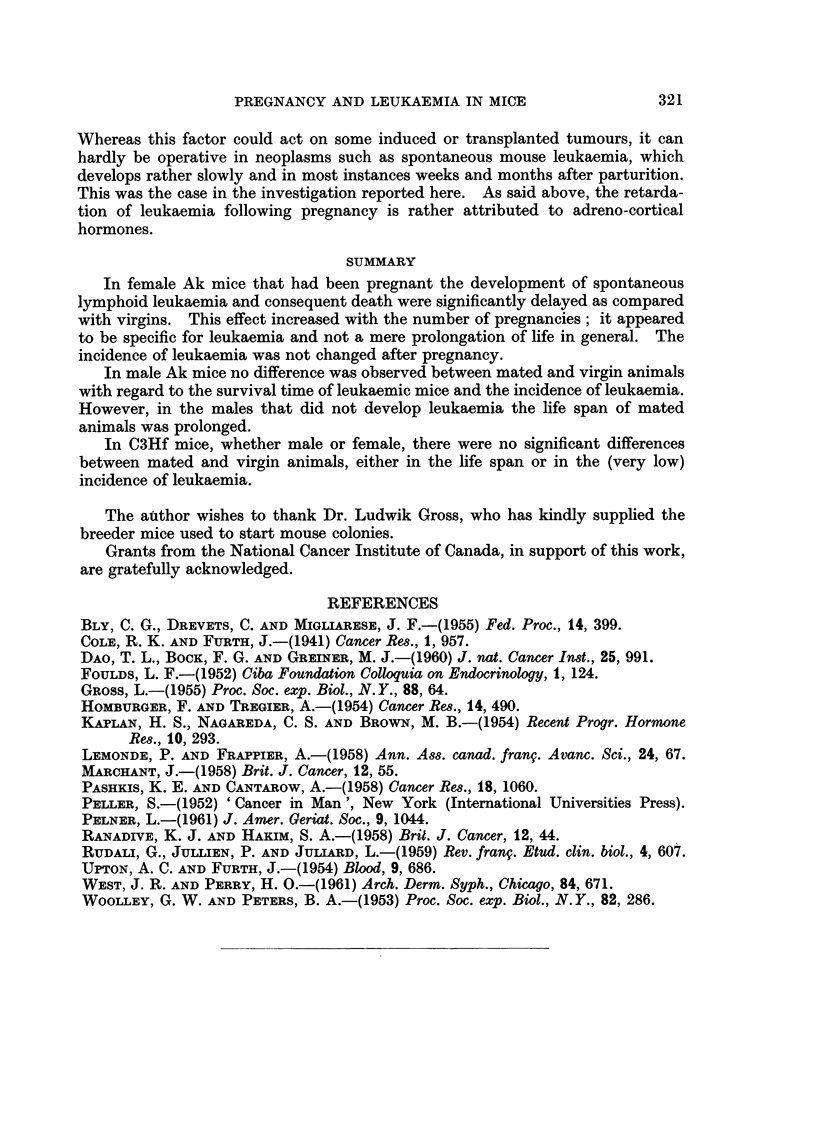# Effect of Pregnancy on Spontaneous Leukaemia in Mice

**DOI:** 10.1038/bjc.1964.37

**Published:** 1964-06

**Authors:** P. Lemonde


### EFFECT OF PREGNANCY ON SPONTANEOUS LEUKAEMIA IN MICE

P. LEMONDE

*From the Institute of Microbiology and Hygiene of the University of Montreal, Canada*

Received for publication March 23, 1964

IN the course of experiments concerned with the action of various physiological and environmental agents on neoplasms, it was deemed advisable to investigate the influence of mating, pregnancy and related factors on the development of spontaneous leukaemia in the mouse.

### MATERIALS AND METHODS

Mice of the inbred Ak strain were used. Our subline is derived, by brother-sister mating, from breeders obtained from Dr. L. Gross in 1955. The incidence of spontaneous lymphomas in these animals is currently 91 per cent.

Mice of the C3Hf strain were used for comparison. Our subline is the offspring of breeders also supplied by Dr. Gross in 1955. These breeders belonged to an inbred line descended from animals received by Gross from J. J. Bittner and freed from the mammary tumour agent by foster nursing, as described by Gross (1955). In our subline, mammary tumours were never seen, whereas the incidence of leukaemia is 2 per cent.

For breeding purposes males and females were placed in cages together, one male to 1–3 females. The females were isolated when pregnant. Sucklings were weaned usually at 24 days of age. If females were mated again it was at least one week after weaning. Mice were mated only when more than 2 months or, as a rule, when less than one year old. Unmated animals were treated in all respects, except for mating, like the former : same diet (Purina Laboratory Chow, water), rooms, kind of cages, etc. All mice were kept under observation until spontaneous death. A few animals that died of accidental causes or on which no autopsy could be performed were excluded from this report.

### RESULTS

Female Ak mice that were mated and became pregnant lived significantly longer than those that remained unmated (Table I). This effect increased with the number of pregnancies : the more litters that were borne, the longer was life prolonged, a finding that is also statistically significant. If the animals that died with leukaemia are considered separately from those without the disease, life prolongation is significant only in the former. It should be noted that, although leukaemia was delayed in mice that had been pregnant, the incidence of the disease remained the same.

TABLE I.—*Effect of Pregnancy on Leukaemia and Life Span in Female Ak Mice*

| Groups | Number of mice | Survival time (avg. days) All mice | Mice with leukaemia | Mice without leukaemia | Incidence of leukaemia (%) |
|---|---|---|---|---|---|
| Unmated | 514 | 260·2± 3·6* | 257·8± 3·3* | 302·7±34·1* | 94·7 |
| Pregnant | 539 | 302·0± 4·2 | 298·3± 3·9 | 368·3±38·0 | 94·8 |
| Once | 367 | 291·8± 4·9 | 290·7± 4·5 | 318·0±54·7 | 95·9 |
| Twice | 145 | 316·2± 8·5 | 308·7± 7·7 | 408·3±57·1 | 92·4 |
| Thrice or more | 27 | 363·2±18·3 | 350·2±17·2 | 525·5± 8·5 | 92·6 |

* Standard error of the mean.

Significance:

Unmated vs. any other group, Pregnant 1 vs. 3 times } all mice and mice with leukaemia : $p < 0\cdot001$

Pregnant 1 vs. 2, or 2 vs. 3 times { all mice : $0\cdot01 < p < 0\cdot02$; mice with leukaemia : $0\cdot001 < p < 0\cdot01$

For reasons explained in the discussion, females that nursed their young normally throughout the lactation period were compared with some that did not nurse because their young were stillborn or died a few hours after birth. No significant difference appeared between the two groups in the survival of leukaemic mice (Table II).

TABLE II.—*Nursing and Leukaemia in Ak Mice*

| Groups | Number of mice | Age at death from leukaemia (avg. days) |
|---|---|---|
| Nursed normally | 416 | 298·2± 4·1* |
| Not nursing | 19 | 340·2±31·1 |

* Standard error of the mean. Not significant : $p > 0\cdot1$.

In males that were mated the life span was also prolonged, but to a lesser degree and less significantly than in females (Table III ; significance between unmated and mated : $0{\cdot}02 < p < 0{\cdot}05$). If the males that became leukaemic are considered separately from those without leukaemia, survival prolongation is significant only in the latter group ($0{\cdot}001 < p < 0{\cdot}01$), so that the overall effect in the males is due to these non-leukaemic animals. There was also a relation between the lengthening of the life span and the number of matings, but it was not quite significant. Here again, as in the females, the incidence of leukaemia was unchanged after mating.

TABLE III.—*Effect of Mating on Leukaemia and Life Span in Male Ak Mice*

| Groups | Number of mice | Survival time (avg. days) All mice | Mice with leukaemia | Mice without leukaemia | Incidence of leukaemia (%) |
|---|---|---|---|---|---|
| Unmated | 549 | 330·4± 4·7* | 322·6± 4·4* | 387·5±21·5* | 88·0 |
| Mated | 464 | 344·8± 5·2 | 325·0±13·8 | 480·6±20·0 | 87·3 |
| Once | 307 | 337·5± 6·2 | 320·9± 5·4 | 458·6±25·1 | 87·9 |
| Twice | 104 | 343·3±10·9 | 316·8± 8·1 | 513·7±38·3 | 86·5 |
| Thrice or more | 53 | 389·6±17·0 | 365·6±14·5 | 524·6±61·1 | 84·9 |

In C3Hf mice, whether male or female, no substantial differences appeared between mated or virgin animals, either in the life span or in the (very low) incidence of leukaemia (Table IV).

TABLE IV.—*Effect of Pregnancy or Mating on Life Span and Leukaemia in C3Hf Mice*

| Groups | Number of mice | Life span (avg. days): All mice | Mice that died of: leukaemia | Mice that died of: other causes | Incidence of leukaemia (%) |
|---|---|---|---|---|---|
| ♀♀ Unmated | 263 | 571·5± 10·6* | 507·0±28·7* | 573·3±10·9 | 2·7 |
| ♀♀ Mated | 282 | 567·3± 9·2 | 438·3±28·7 | 570·1± 9·3 | 2·1 |
| ♂♂ Unmated | 476 | 566·7± 6·2 | 557·8±29·6 | 566·8± 6·3 | 1·7 |
| ♂♂ Mated | 425 | 557·4± 5·4 | 529·7±19·7 | 558·1± 5·6 | 2·4 |

* Standard error of the mean.

### DISCUSSION

The results show that a delay in the development of leukaemia and a consequent retardation of death are associated with pregnancy in female Ak mice. This effect is rather specific for leukaemia, and not a prolongation of the life span in general, since the difference between unmated and pregnant females is significant only in mice that died with leukaemia ; this view is further supported by the fact that no such effect of pregnancy on survival time occurred in the low-leukaemic strain C3Hf. The incidence of leukaemia was the same in all females, whether virgins or mothers ; in other words, leukaemia was delayed, but not prevented. This unchanged incidence of leukaemia after pregnancy in Ak mice was previously noted by Cole and Furth (1941). Preliminary findings on leukaemia retardation by pregnancy were reported earlier (Lemonde and Frappier, 1958). In the present paper these former results are confirmed, extended and compared with observations in males and in C3Hf mice. A prolongation, by breeding, of the latent period before leukaemogenesis in AkR mice was also reported by Rudali, Jullien and Juliard (1959).

In Ak males the prolongation of life associated with sexual activity is an effect on life span in general rather than on leukaemia ; it does not appear in leukaemic animals ; it is brought about by the males that escaped leukaemia and died later of other causes, mainly degenerative diseases. It seems to be peculiar to Ak males, as it is not seen in mice of the C3Hf strain.

Is leukaemia retardation in females due to pregnancy or to sexual activity and mating? This question remains open. Mating without gestation can be achieved, for example by ligating *vasa deferentia* in males, but pseudo-pregnancy would still occur and confuse the picture. However, let us recall by comparison, although the situation is of course different, that sexual activity had no effect on leukaemia in males, which suggests that gestation rather than sexual activity impedes leukaemia in females.

It may be wondered whether leukaemia retardation is associated with pregnancy only or also with lactation. Breeding without lactation can be effected by removing the newborn from the mother just after birth. This was not done here because removal of the young and interruption of lactation could, in themselves, influence leukaemia ; in addition, no control group of lactation without pregnancy is feasible, except through complicated procedures that might *per se* affect the issue. However, this situation occurred naturally in some females whose young were stillborn or died shortly after birth. If these females are compared with the mothers that suckled their young normally, the survival of leukaemic mice was not significantly different between the two groups ; there was even a tendency for nursing females to die earlier (Table II). This provides an indication that pregnancy rather than lactation afforded the delaying effect on leukaemia.

With respect to the mechanism through which leukaemia is hindered in female mice that have borne young, several possibilities may be considered. It is unlikely that oestrogens are responsible, since they are known to promote, enhance and accelerate leukaemia. As regards progesterone, this hormone has not been reported to have any effect on leukaemia (Kaplan, Nagareda and Brown, 1954 ; Rudali *et al.*, 1959). An experiment concerning its influence on spontaneous leukaemia was carried out with AkR mice and it was found ineffective (Rudali *et al.*, 1959). It is possible that adreno-cortical hormones, of the corticosterone type, which are secreted in greater amounts during pregnancy, are responsible for the observed retardation of leukaemia. These hormones are known to inhibit or delay leukaemia clinically, as well as experimentally in mice (Woolley and Peters, 1953 ; Upton and Furth, 1954 ; Kaplan *et al.*, 1954).

Observations reported in the literature about the relations between gravidity and cancer in general are not quite consistent. Many authors have described tumour inhibition resulting from pregnancy; some have found no effect and a few have reported an aggravation. However, the majority clearly stand for inhibition or retardation of cancer by pregnancy (Pelner, 1961). With regard to tumours induced by chemical carcinogens, only mammary carcinomas, to my knowledge, have been studied in connection with pregnancy, with variable results (Marchant, 1958 ; Ranadive and Hakim, 1958 ; Dao, Bock and Greiner, 1960). Mammary tumours are not very suitable for showing an action of pregnancy on cancer in general, since normal breast tissue is itself strongly influenced by the factors that influence gestation. With transplanted tumours, an inhibition or regression was reported in most cases (Homburger and Tregier, 1954; Bly, Drevets and Migliarese, 1955 ; Pelner, 1961), though no effect or enhancement was noted (Pashkis and Cantarow, 1958 ; Pelner, 1961). Little experimental work has been done with spontaneous tumours. Foulds (1952) described spontaneous breast carcinomas that regressed at the end of pregnancy or after parturition in mice ; Haddow and others mentioned similar cases in a discussion of Fould's paper. Other instances are quoted by Pelner (1961). In women the actual incidence of malignant diseases was said to be lower than the expected incidence in pregnant individuals (Peller, 1952). Clinical papers on gestation and cancer do not present concordant findings. Nevertheless, retardation of neoplasms was claimed to be the most frequent result (Pelner, 1961). Recently, pregnancy was reported to be effective in clearing multiple warts without recurrences (West and Perry, 1961).

Among the factors of cancer inhibition by pregnancy, various authors have considered oestrogens, gonadotrophins or progesterone as being instrumental. Regarding progesterone, investigators who have studied this hormone experimentally have failed to find any effect on leukaemia, as already mentioned, or on other tumours (Bly *et al.*, 1955). Some authors have explained tumour inhibition as resulting from a competition for nutrients between foetuses and tumours.

Whereas this factor could act on some induced or transplanted tumours, it can hardly be operative in neoplasms such as spontaneous mouse leukaemia, which develops rather slowly and in most instances weeks and months after parturition. This was the case in the investigation reported here. As said above, the retardation of leukaemia following pregnancy is rather attributed to adreno-cortical hormones.

### SUMMARY

In female Ak mice that had been pregnant the development of spontaneous lymphoid leukaemia and consequent death were significantly delayed as compared with virgins. This effect increased with the number of pregnancies ; it appeared to be specific for leukaemia and not a mere prolongation of life in general. The incidence of leukaemia was not changed after pregnancy.

In male Ak mice no difference was observed between mated and virgin animals with regard to the survival time of leukaemic mice and the incidence of leukaemia. However, in the males that did not develop leukaemia the life span of mated animals was prolonged.

In C3Hf mice, whether male or female, there were no significant differences between mated and virgin animals, either in the life span or in the (very low) incidence of leukaemia.

The author wishes to thank Dr. Ludwik Gross, who has kindly supplied the breeder mice used to start mouse colonies.

Grants from the National Cancer Institute of Canada, in support of this work, are gratefully acknowledged.

### REFERENCES

Bly, C. G., Drevets, C. and Migliarese, J. F.—(1955) *Fed. Proc.*, **14**, 399.
Cole, R. K. and Furth, J.—(1941) *Cancer Res.*, **1**, 957.
Dao, T. L., Bock, F. G. and Greiner, M. J.—(1960) *J. nat. Cancer Inst.*, **25**, 991.
Foulds, L. F.—(1952) *Ciba Foundation Colloquia on Endocrinology*, **1**, 124.
Gross, L.—(1955) *Proc. Soc. exp. Biol., N.Y.*, **88**, 64.
Homburger, F. and Tregier, A.—(1954) *Cancer Res.*, **14**, 490.
Kaplan, H. S., Nagareda, C. S. and Brown, M. B.—(1954) *Recent Progr. Hormone Res.*, **10**, 293.
Lemonde, P. and Frappier, A.—(1958) *Ann. Ass. canad. franç. Avanc. Sci.*, **24**, 67.
Marchant, J.—(1958) *Brit. J. Cancer*, **12**, 55.
Pashkis, K. E. and Cantarow, A.—(1958) *Cancer Res.*, **18**, 1060.
Peller, S.—(1952) ‘Cancer in Man’, New York (International Universities Press).
Pelner, L.—(1961) *J. Amer. Geriat. Soc.*, **9**, 1044.
Ranadive, K. J. and Hakim, S. A.—(1958) *Brit. J. Cancer*, **12**, 44.
Rudali, G., Jullien, P. and Juliard, L.—(1959) *Rev. franç. Etud. clin. biol.*, **4**, 607.
Upton, A. C. and Furth, J.—(1954) *Blood*, **9**, 686.
West, J. R. and Perry, H. O.—(1961) *Arch. Derm. Syph., Chicago*, **84**, 671.
Woolley, G. W. and Peters, B. A.—(1953) *Proc. Soc. exp. Biol., N.Y.*, **82**, 286.